# Oral findings in postmenopausal women attending dental hospital in Western part of India

**DOI:** 10.4317/jced.50928

**Published:** 2013-02-01

**Authors:** Patil Santosh, Sinha Nidhi, Kaswan Sumita, Rahman Farzan, Doni Bharati, KP Ashok

**Affiliations:** 1 Dept of Oral medicine and radiology, Jodhpur Dental College, Jodhpur National University, Jodhpur (Raj). India; 2 Dept of Conservative dentistry and endodontics, Jodhpur Dental College, Jodhpur National University, Jodhpur (Raj). India; 3 Dept of Oral Pathology and Microbiology, Jaipur Dental College and Hospital, Jaipur (Raj), India; 4 Dept of Oral medicine and radiology, NIMS, Jaipur (Raj), India; 5 Dept of Periodontics, Vyas Dental College and Hospital, Jodhpur, India

## Abstract

Objectives: To know the nature, incidence and severity of oral manifestations occurring in postmenopausal women. Study design: Oral changes were observed in 365 postmenopausal women and 365 age matched male individuals attending the department of Oral Medicine and Radiology. The patients were asked about complaints of dry mouth, taste and breath changes, mucosal and facial pain and were examined for oral changes such as ulceration, white and red lesions. The results obtained from the study were then correlated with various other similar studies. Results: The important oral findings in postmenopausal women were mucosal burning/pain (25.8%), dry mouth (27.1%), altered taste (3.6%), altered breath (6.3%) and facial pain (3.6%). Oral submucous fibrosis (OSMF) was significantly more common in males (5.5%) as compared to postmenopausal females (1.9%). Conclusion: Results from the present study reveal that oral symptoms are common problems in postmenopausal women. Postmenopausal patients showed significantly more oral changes than the control. These changes could be related to the hormone alterations. Therefore, dentists need to refer postmenopausal women with oral symptoms to a gynaecologist for more careful examinations and medical interventions if necessary.

** Key words:**Menopause; postmenopause; xerostomia; pallor; oral changes.

## Introduction

Menopause in women is a physiological state that gives rise to adaptive changes at both systemic and oral level. Menopause literally means “without estrogen” and is, by definition, the time at which cyclic ovarian function, as manifested by menstruation, ceases. The critical period in which menstruation ceases, defines the term ‘Climacterium’, which is often used in reference to menopause. However, menopause is not synonymous to climacterium. In general, menopause should be considered as the date of the last menstruation and as such it represents a brief and defined period of time (an interruption of 12 months), while climacterium implies a much longer period involving a series of events such as the loss of female reproductive capacity and the occurrence of important changes in sex hormone secretion. These events induce major modifications in the genital apparatus as well as in other areas of the body (1).

Research community has paid limited attention in context to menopause, with information mostly based on clinical impressions or subjective, anecdotal case reports. Various studies have suggested that menopause initiates a host of physiologic changes that include endocrinological alterations and atrophy of tissues lining the vagina and in the urinary tract. Additionally, decreased estrogen levels increases the risk of developing heart disease and osteoporosis in menopausal and postmenopausal women (2-5).

Earlier reports on the various oral manifestations of menopause emphasized on cytological studies of the oral mucosa, gingiva and vagina of postmenopausal women and reported tissue changes (6-10). Additional studies, later supported correlations between estrogen deficiency and oral changes seen during menopause (11-13). However, local irritating factors rather than hormonal influences according to Bercovici et al. (14) lead to the oral discomfort in postmenopausal women.

Estrogen deprivation arising as a result of menopause along with age-related factors disproportionately increases the risk of developing osteoporosis, cardiovascular diseases like myocardial infarct, stroke, Alzheimer’s disease and diseases of the oral cavity. To alleviate the uncomfortable symptoms associated with estrogen deficiency and to prevent some of the chronic illnesses common to post menopausal women, hormone replacement therapy ( HRT- estrogen or estrogen and progestin) often is prescribed on a short-term and a long-term basis (15). In addition to the more general manifestations of menopause however, oral symptoms are also found. Along with the physiological aging of the oral tissues, the hormone changes that take place in menopausal women are responsible for the alterations observed within the oral cavity (16). Dental practitioner today is expected to encounter increasing numbers of postmenopausal women in parallel to longer life expectancies. The crucial issue therefore, is to be aware of the possible changes associated with menopause that can lead to more serious health problems, although these changes may not be uncomfortable to the patient. Therefore, by recognizing and treating the oral complications seen in association with menopause, the dental practitioner can play a pivotal role in the management of postmenopausal women. With this background, this study is designed to evaluate the oral changes in postmenopausal women

## Material and Methods

This was a cross sectional, descriptive-analytic study carried out in Jodhpur Dental College General Hospital. Ethical clearance was obtained from the Institutional Ethical Committee to undertake the study. A total of 730 aged individuals aging from 54 to 70 years, with informed consent, were selected by non-probable sample from those attending for routine dental complaints in the year 2011. The case group consisted of 365 post menopausal women with a mean age of 62.73 ± 4.73 years. The control group included 365 age-matched men with a mean age of 62.23 ± 4.27 years ( Table 1).

**Table 1:**
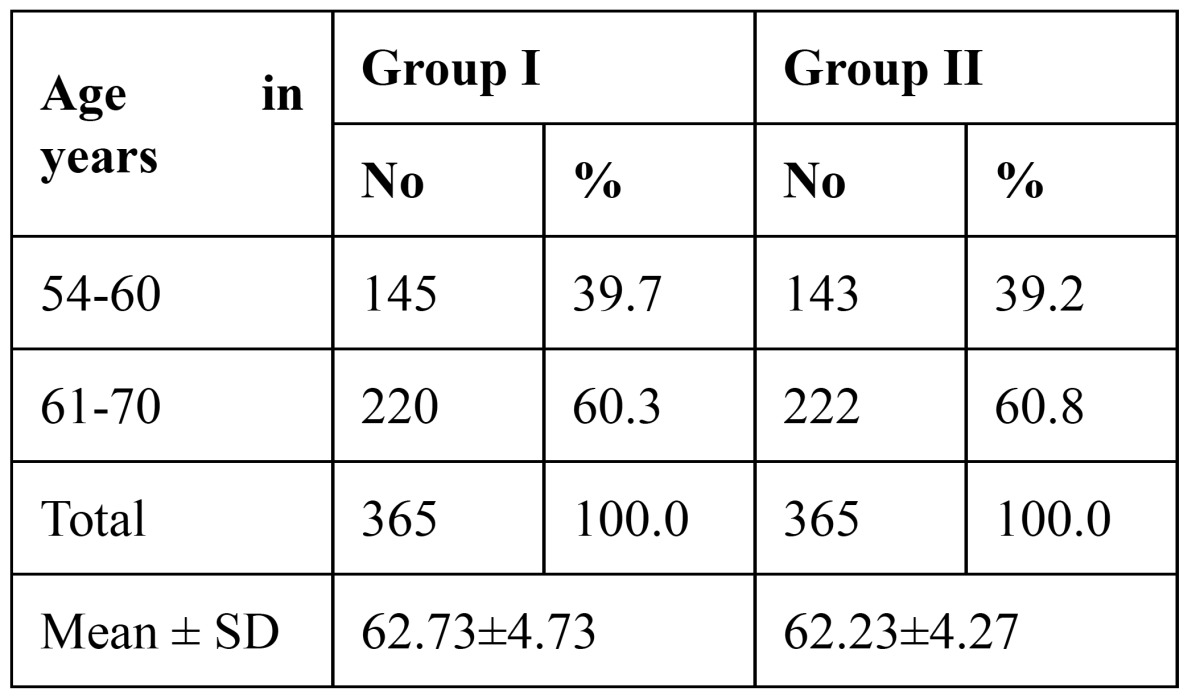
Age distribution of subjects studied.

All patients taking part in the study were required to fulfill the following inclusion criteria:

• Female,

• Aged 50 or over,

• Cessation of menstruation for 12 months,

• Hormonal analysis compatible with menopause.

Exclusion criteria included the following:

• Local inflammation,

• Focal infection and fibrosis of the major salivary glands,

• Sjögren syndrome,

• Mikulicz disease, 

• Dehydration,

• Autoimmune diseases,

• Post radiotherapy changes,

• Chemotherapy.

The patients were asked about dry mouth, taste and breath changes, mucosal/facial pain, burning sensation and they were examined for oral changes such as ulceration, white and red lesions among others. A performa was framed and all the relevant information of each patient was recorded in the same.

## Statistical Analysis

SPSS (Statistical Package for the Social Sciences) program was used for statistical analysis of the collected data. c2 analysis was utilized to examine the significance of the differences in means and distribution of categorical variables between groups. A p<0.05 level of significance was chosen.

## Results

The mean number of years elapsed since menopause in the postmenopausal women was found to be 7.88 ± 4.41 years ( Table 2). Group II had more habits of areca nut and tobacco usage as opposed to Group I ( Table 3), sug-gestive of higher incidence of oral submucous fibrosis in males (5.5%) ( Table 4), which was found to be significant with p=0.011. Clinical manifestations seen in postmenopausal women, such as mucosal burning/pain (25.8%), breath change (6.3%), facial pain (3.6%) and dry mouth (27.1%) was highly significant in contrast to those seen in the similar age-matched males ( Table 4). No significant difference was however seen in relation to taste changes, presence of white lesions and ulcerative lesions in both the groups. There were no red lesions seen in either the postmenopausal females or the age-matched males.

**Table 2:**
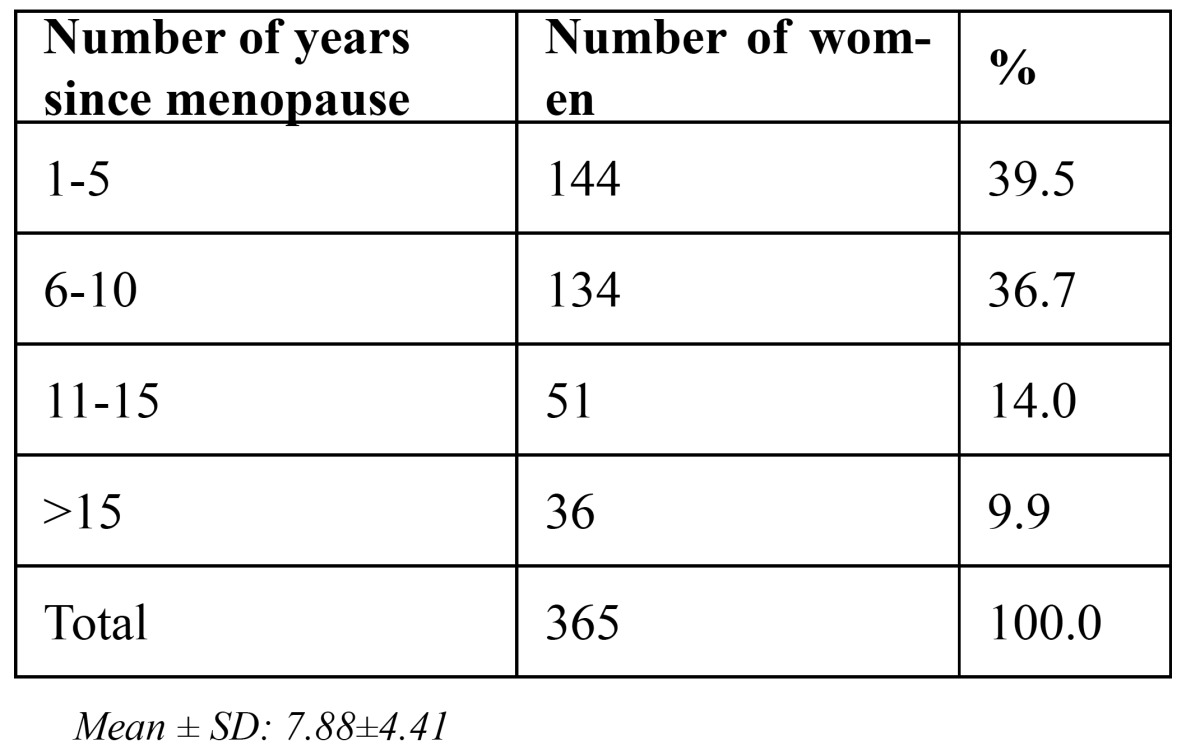
Number of years of menopause of women studied.

**Table 3:**
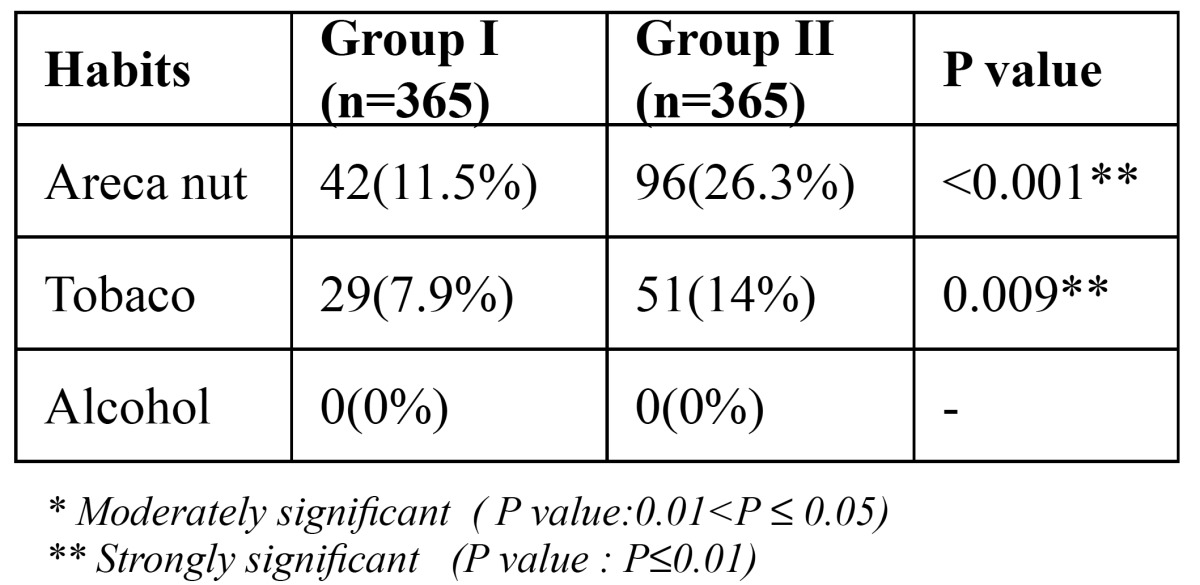
Habits.

**Table 4:**
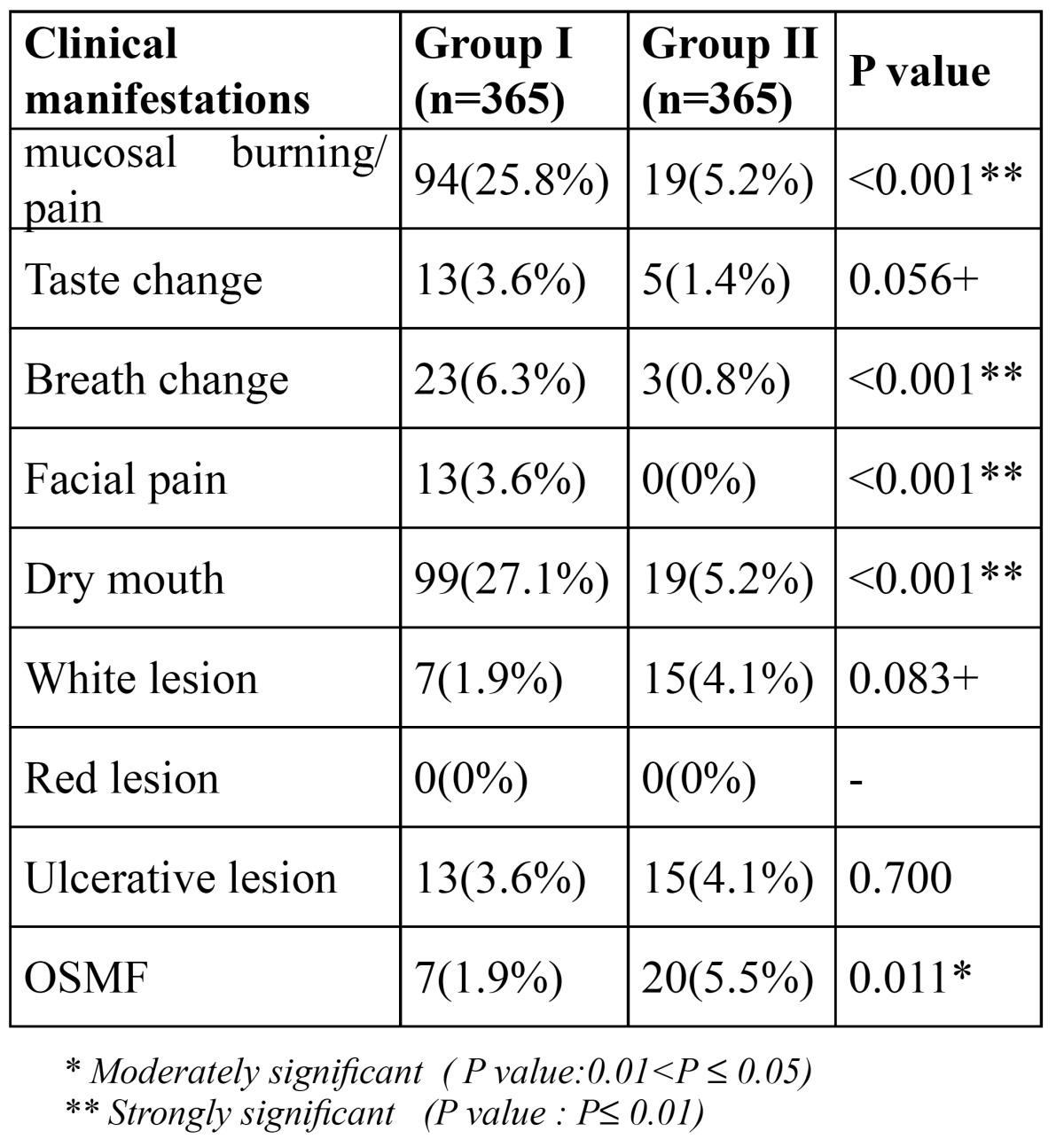
Clinical manifestations.

## Discussion

Menopause is found to be associated with significant adverse changes in the oro-facial complex along with other general changes. One of the major oral problems in these patients is burning mouth syndrome, which is characterized by intense pain and a spontaneous burning sensation affecting mainly the tongue and sometimes the lips and gums. However, despite the intense symptoms of pain and burning sensation, no appreciable organic lesions are generally observed, as seen in the present study. In addition to burning mouth, these patients refer alterations in taste (dysgeusia) and breath, dry mouth, swallowing difficulties and facial or dental pain (17). Wardrop et al. (18) in a study of postmenopausal women aged 30 to 63 years and receiving treatment for control of the symptoms, found 33% women with oral manifestations including burning mouth, though no evidence of any organic lesions capable of accounting for such alterations were observed. They further noted the prevalence of oral discomfort in postmenopausal women to be 46%, when compared to 6% for premenopausal women. Women account for 80% of burning mouth syndrome patients with a marked (3:1) female predominance. The pain began from 3 years before to 12 years after onset of menopause. This gender difference increases with age, as also seen in the present study where the ratio increased to 5:1 suggesting that menopause plays an important role in the incidence of glossodynia. Mean patient age for the onset of burning mouth syndrome is 50-60 years. Certain micro-organisms such as Candida albicans, Staphylococci, Streptococci and various anaerobes have also been suggested as the etiological factor of burning mouth syndrome, along with xerostomia (associated to Sjogren’s syndrome, anxiety and medication), anemia (Pernicious or Iron deficiency), nutritional disorders related to vitamin B complex or iron, diabetes mellitus, climacteric hypoestrogenemia per se, certain mechanical factors (abnormal oral habits, chronic denture-induced irritation) and other idiopathic factors (19). Psychological disorders, including depression, anxiety, phobia for severe illnesses such as cancer and other psychogenic alterations appear to play a fundamental role (20). With regards to this, Trikkas et al. (21) while examining in a group of 25 subjects with burning mouth syndrome in the age range of 52-75 years, found disorders such as hostility- particularly in introverted individuals, anxiety, phobias and depression to form an integral component of the psychological profile of patients with burning mouth syndrome. Approximately two-thirds of the study subjects who presented oral symptoms but no evident clinical manifestations were seen to improve with hormone replacement therapy. The diagnosis of burning mouth syndrome is based on the compilation of a detailed case history, the absence of findings in the physical examination and laboratory tests and the exclusion of other possible oral disorders. In general, a clinical diagnosis is established without difficulty, though the factors underlying the symptoms are either difficult or impossible to identify (19). Xerostomia is another frequent symptom seen during menopause with 27.1% postmenopausal females as compared with only 5.2% age-matched males showing the symptom in the present study. Postmenopausal women have decreased unstimulated and stimulated submandibular and sublingual salivary gland flow compared with premenopausal women, a finding unrelated to any medication effect (15). In this context, although an association to menopause exists, the decreased salivary flow is known to be related with age, though not always necessary. Evaluation of the salivary function in postmenopausal women to explain the association between dry mouth and the climacterium, has been attempted in a number of studies. Some authors have found the decreased salivary flow to be associated to menopause, while a few others have been unable to identify changes in either salivary volume or composition (22,23). No significant differences in salivary flow volume in premenopausal women versus postmenopausal females were observed in the longitudinal study conducted by Ship et al (13). However, important differences were recorded in regards to salivary flow between postmenopausal women receiving estrogen therapy and postmenopausal females without any such treatment. Hence, a conclusion was drawn by these authors that none of the healthy women participating in their study showed signs of burning mouth, xerostomia or alterations in salivary output. In a case-control study of 46 postmenopausal women (38 with dry mouth sensation and 38 without xerostomia) carried out by Agha-Hosseini (24), a negative correlation was however found between the severity of dry mouth sensation and the salivary concentration of 17-beta-estradiol. Significant differences (p<0.05) however among the same age males and postmenopausal females was observed in the results of this study. Ben Aryenh et al. (25) found 45% of menopausal women without general symptoms to suffer oral manifestations, while 60% of the patients with general features also had oral problems. Salivary flow or composition between the two groups showed no significant changes. Nevertheless, the salivary IgA concentration and total proteins were significantly higher among these subjects when compared with healthy young women, a phenomenon that may be attributed to the psychological stress to which these patients may be exposed. The paucity of saliva has been implicated as a cause of increased dental caries, and may be responsible for the increased prevalence of oral dysesthesia and taste alterations, as observed in this study. The xerostomia is thought to be responsible for an increase in caries, periodontal disease and oral candidiasis (15).

Dry mouth not only is annoying, but may lead to yeast infections, dental caries, mouth ulcers, and oral malodor because saliva functions to wash away food debris, plaque, carbohydrates, and helps prevent new plaque buildup along with remineralization of the teeth and combating harmful micro-organisms (16). Dry mouth is not the only cause for oral malodor seen in postmenopausal women. Various other contributing factors such as those related to the digestive system, increased incidence of caries and periodontal problems, yeast overgrowth due to inability to maintain good denture hygiene, sinusitis, which are shown to afflict older people more than the younger are responsible for bad breath as seen in older females approaching towards menopause. Bad breath may also be due to tobacco use as also seen in women now-a-days. This is evident in the present study as 29 women had tobacco related habits and of them 23 women complained of bad breath. Nutritional status is also important during menopause as nutritional condition may have a direct effect on the chemosensory function, which in turn would induce changes in dietary habits. Individuals with loss of sensitivity to sweet tastes may sweeten foods with potentially serious consequences, especially for those with diabetes mellitus, cardiac disease or obesity. A significant reduction in sucrose perception and palatal sensitivity in postmenopausal women was noted by Delibasi et al. (26) in their study7 (35%) of the female patients noticed alteration in taste perception during the postmenopausal period. The taste changes in the present study was however not significant (p>0.05) when compared with same age control males. Incidence of OSMF is significantly less in female patients (1.9%) when compared to males (5.5%), this may be due to less number of women with the habits of areca nut and tobacco usage. The white lesion in the present study were lichen planus like lesions which may be developed secondary to the medications taken by patients and leukoplakia which may be developed secondary to usage of tobacco, hence these findings were not related to the hormonal changes seen in association to menopause.

## Conclusion

To deliver high quality care, dental practitioners need to be knowledgeable about menopause and its oral manifestations as a possible risk factor for increasing oral health problems. Current demographic trends in the Indian female population underscore this need. For example, the menopausal patient who comes to the dental clinic with complains of oral discomfort or loose teeth may not understand the etiology of her dental concerns. A knowledgeable dental practitioner therefore, could advise that the conditions are possibly menopause-related and provide her with more comprehensive patient education, periodontal debridement, and use of daily home rinses. Dental practitioners can play a vital role in meeting the oral health needs of the menopausal patient by early diagnosis, treatment planning, and patient education. Further research is however indicated to corroborate the findings of this study, determine causes, and investigate to improve the knowledge levels to a further extent. A national survey of dental hygiene programs is hereby suggested to help determine whether menopause or its oral effects are included in curricula. Provision of optimal patient care is the primary issue and is paramount to the dental practitioner.
